# Introduction to Dermatology

**Published:** 1916-12

**Authors:** 


**Introduction to Dermatology.** By Norman Walker, M.D., F.R.C.P. Sixth Edition. Pp. xiv., 363. Edinburgh and London: William Green & Son. 1916. Price 15s. net.

This highly successful treatise on skin diseases has reached its sixth edition, and is undoubtedly a great boon to medical students. The editor has taken a wise lead in advising them to call as few cases as possible "eczema." He has given them means fcr exterminating a large number of cases which were formally called by this name.

Although the book is small as compared with most others on this subject, it contains all the valuable information which is really necessary, and is the outcome of powers of concentration unusually developed.

The book is lavishly illustrated with really good pictures, which must prove very helpful. The articles on dermatitis venenata and dermatitis autophyta are cleverly done, and we consider reflect great credit on a difficult subject of dermatology. Perhaps the author has laid himself unduly open to attack when he states: "There is no room for doubt that certainly in the earlier stages exposure to radium is the most satisfactory method of treating rodent ulcer." We are of opinion that most dermatologists think they get better results from a full pastille dose or more of X-rays. Some workers first apply carbon dioxide snow, and then after a few days X-rays.

